# Sequential accumulation of phosphorylated tau species in early Alzheimer's disease

**DOI:** 10.1002/alz70855_102149

**Published:** 2025-12-23

**Authors:** Arline Faustin, Aysha Strobbe, Kaleah Balcomb, Evgeny Kanshin, Tomas Kavanagh, Sian Genoud, Geoffrey Pires, Manor Askenazi, Beatrix Ueberheide, Thomas Wisniewski, Eleanor Drummond

**Affiliations:** ^1^ NYU Grossman School of Medicine, New York, NY, USA; ^2^ The University of Sydney, Sydney, NSW, Australia; ^3^ University of Sydney, Sydney, NSW, Australia; ^4^ Biomedical Hosting LLC, Arlington, MA, USA; ^5^ New York University Grossman School of Medicine, New York, NY, USA

## Abstract

**Background:**

Emerging studies suggest that phosphorylation of specific tau residues may occur in a sequential manner in Alzheimer's disease (AD). This has important therapeutic and biomarker implications. While initial studies show promise, our understanding of which type of pTau that accumulates earliest in AD is still limited. Therefore, the aim of this study was to perform a detailed proteomic and neuropathological study of pTau accumulation in early AD.

**Method:**

Localized proteomics was used to profile protein changes in human post‐mortem brain tissue from early AD cases with pathology, but no cognitive impairment (preclinical AD cases; *n* = 20), cases with pathology and mild cognitive impairment (MCI; *n* = 20) and age‐matched control cases (*n* = 20). Protein changes were assessed in the vulnerable inferior temporal cortex and the comparatively resistant primary visual cortex from each case. Validation studies were performed on the same tissue using immunofluorescence to assess abundance and localization of pTau181, pTau217, pTau231, and pTau202/205.

**Result:**

>3,800 proteins were quantified using localized proteomics. Midkine emerged as the most significantly altered protein in early AD in both the temporal cortex and visual cortex. Proteomics also identified unique pTau species that correlated with vulnerability to disease. For example, pTau217 best correlated with disease vulnerability, with greater abundance in temporal cortex than visual cortex and in MCI > preclinical AD > controls, while pTau181 and pTau202 were more consistently observed in all groups, including controls. The minimal amount of pTau in the visual cortex in early AD allowed analysis of the sequential accumulation of specific pTau species using immunofluorescence. Neuritic pTau181 was the earliest tau accumulation we observed. As tau pathology accumulated, pTau217 and pTau202/205 pathology were next observed, followed by pTau231. Each type of pTau showed similar aggregate morphology, with neuritic pathology being most prevalent, followed by tangles and plaque‐associated neurites as disease progressed.

**Conclusion:**

Our results suggest that pTau accumulation in early AD may occur in a staged manner in AD, with pTau181 accumulation in neurites being observed before other pTau species. Additionally, our proteomics results highlight the potentially important role for midkine in early AD.

